# ﻿On *Ypsolopha* micromoths (Lepidoptera, Ypsolophidae) associated with *Adesmia* shrubs (Fabaceae) in the arid western slope of the central Andes

**DOI:** 10.3897/zookeys.1195.116134

**Published:** 2024-03-14

**Authors:** Héctor A. Vargas

**Affiliations:** 1 Departamento de Recursos Ambientales, Facultad de Ciencias Agronómicas, Universidad de Tarapacá, Arica, Chile Universidad de Tarapacá Arica Chile

**Keywords:** Arid environments, DNA barcoding, oligophagy, phytophagous larvae, South America

## Abstract

*Ypsolopha* Latreille, 1796 (Lepidoptera, Ypsolophidae) is a genus comprised mostly of Holarctic micromoth species with a fairly broad range of larval hosts (e.g. Aceraceae, Rosaceae, and Fagaceae). The only previous record of herbivory on a representative of the South American genus *Adesmia* DC. (Fabaceae) was based on the discovery of *Ypsolophamoltenii* Vargas, 2018 larvae feeding on *Adesmiaverrucosa* Meyen in the Andes of northern Chile. Further surveys revealed *Adesmiaatacamensis* Phil. as another host for *Y.moltenii*, and *Adesmiaspinosissima* Meyen as the single host of *Ypsolopha* sp. The genetic distance between DNA barcodes of the two micromoth species was 7.9–8.1% (K2P). These results suggest narrow host ranges for *Adesmia*-feeding *Ypsolopha* and highlight the need to further explore the taxonomic diversity of these micromoths in other South American environments.

## ﻿Introduction

The widespread and highly diverse micromoth genus *Ypsolopha* Latreille, 1796 (Lepidoptera, Yponomeutoidea, Ypsolophidae) includes more than 160 described species, most of which occur in the Nearctic and Palearctic regions (Jin et al. 2013; Ponomarenko 2020; Sachkov and Zolotuhin 2020; Corley and Ferreira 2021). Only eight Neotropical species have been recorded so far, but recent discoveries suggest that this apparent low diversity could be due to a lack of sampling effort and taxonomic studies (Vargas 2018, 2021). Larvae of *Ypsolopha* generally build a loose silk web on their host plants while feeding, and a dense silk cocoon with a narrow apical opening for pupation. Although the host plants are only partially known, the available records suggest that species are oligophagous or polyphagous (Dugdale et al. 1998; Anikin et al. 2006; Jin et al. 2013; Sohn et al. 2013; Akulov et al. 2018).

The South American plant genus *Adesmia* DC. (Fabaceae) includes nearly 230 species classified in two subgenera, *Adesmia* and *Acanthadesmia*, and 45 series (Burkart 1967). Despite the remarkable species richness, this genus remained unknown as a host for *Ypsolopha* until the recent discovery of larvae of *Ypsolophamoltenii* Vargas, 2018 feeding on the shrub *Adesmiaverrucosa* Meyen, a member of the subgenus Adesmia, on the arid western slope of the Andes of northern Chile (Vargas 2018). Subsequent surveys in this mountainous area revealed larvae of the congeneric *Ypsolophachicoi* Vargas, 2021 associated with the shrub *Muehlenbeckiafruticulosa* (Walp.) Standl. (Polygonaceae) (Vargas 2021), but no additional field observations of *Adesmia*-feeding *Ypsolopha* micromoths have been documented.

**Figures 1–4.**
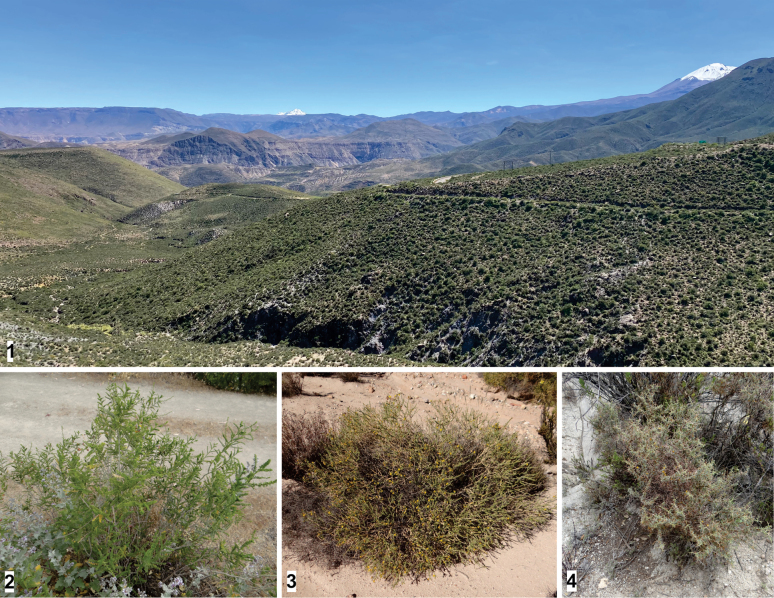
Shrubs of the South American genus *Adesmia* DC. (Fabaceae) co-occurring in the Andes of northern Chile **1** typical landscape near Socoroma Village at about 3400 m elevation **2***Adesmiaverrucosa* Meyen **3***Adesmiaatacamensis* Phil. **4***Adesmiaspinosissima* Meyen.

The distribution ranges of some members of the two subgenera of *Adesmia* overlap in northern Chile (Ulibarri 1986; Macaya-Berti and Teillier 2022), such as in the surroundings of the type locality of *Y.moltenii*, where *A.verrucosa* co-occurs with *Adesmiaatacamensis* Phil. (subgenus Adesmia) and *Adesmiaspinosissima* Meyen (subgenus Acanthadesmia), indicating the possibility of a wider host range for this micromoth (Figs 1–4). The aim of this study was to explore the interaction between *Ypsolopha* micromoths and *Adesmia* shrubs native to this arid, high-elevation area.

## ﻿Material and methods

Surveys were performed near Murmuntani (18°20'43"S, 69°33'06"W), Socoroma (18°16'42"S, 69°34'15"W), Putre (18°13'01"S, 69°33'39"W) and Zapahuira (18°19'22"S, 69°35'18"W), at about 3400–3700 m elevation on the western slope of the Andes of the Parinacota Province of northern Chile between April 2018 and April 2022. This area has a tropical xeric climate, with seasonal rains concentrated mainly in summer (Luebert and Pliscoff 2006). Larvae of *Ypsolopha* were searched for on at least 50 plants of the following native species of Fabaceae: *A.atacamensis*, *A.spinosissima*, Daleapennellii (J.F. Macbr.) J.F. Macbr. var. chilensis Barneby, *Lupinusoreophilus* Phil., and Sennabirostris (Dombey ex Vogel) H.S. Irwin & Barneby var. arequipensis (Meyen ex Vogel) H.S. Irwin & Barneby. The collected larvae were brought to the laboratory in plastic vials with parts of the respective host plants and reared to obtain adults. In order to provide taxonomic identifications, the abdomens of adults were removed for dissection of the genitalia using standard procedures. In addition, larvae of the only two species of *Ypsolopha* previously recorded in the study area, *Y.chicoi* and *Y.moltenii*, were collected from their hosts for comparison; some were placed in ethanol 95% and kept at −20 °C to be used for DNA extraction, while the others were reared to obtain adults. Vouchers and slides containing genitalia are deposited in the “Colección Entomológica de la Universidad de Tarapacá” (**IDEA**), Arica, Chile.

Genomic DNA was extracted from adult legs or larvae using the QIAamp Fast DNA Tissue Kit (Qiagen). The primers LCO1490 and HCO2198 (Folmer et al. 1994) were used for PCR amplification and sequencing of the barcode region (Hebert et al. 2003) with a program of 5 min at 94 °C, 35 cycles of 30 s at 94 °C, 30 s at 47 °C, 1 min at 72 °C, and a final elongation step of 10 min at 72 °C. DNA purification, amplification, and sequencing were performed at Macrogen Inc. (Seoul, South Korea). MEGA v. 11 (Tamura et al. 2021) was used for sequence alignment with the ClustalW method, to assess the genetic distance using the Kimura 2-Parameter (K2P) method and to build a neighbor-joining tree with 1,000 bootstrap replicates.

## ﻿Results

**Figures 5, 6.**
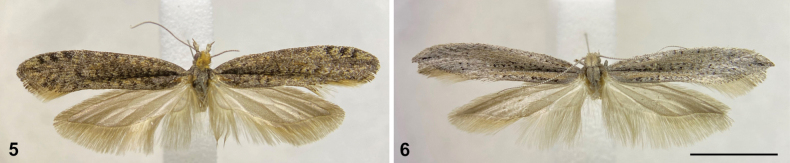
Micromoths of the genus *Ypsolopha* Latreille, 1796 (Lepidoptera, Ypsolophidae) from the Andes of northern Chile, representing new host records of the South American genus *Adesmia* DC. (Fabaceae) **5** male of *Ypsolophamoltenii* Vargas, 2018 reared from larvae collected on *Adesmiaatacamensis* Phil., dorsal view, abdomen removed **6** male of *Ypsolopha* sp. reared from larvae collected on *Adesmiaspinosissima* Meyen, dorsal view, abdomen removed. Scale bar: 5 mm.

The surveys revealed larvae of *Ypsolopha* on *A.atacamensis* and *A.spinosissima* but not on *D.p.chilensis*, *L.oreophilus*, or *S.b.arequipensis*. Two females and two males of *Y.moltenii* were reared from the larvae collected on *A.atacamensis*, and two males of *Ypsolopha* sp. from the larvae collected on *A.spinosissima* (Figs 5, 6). In addition, adults of *Y.chicoi* and *Y.moltenii* were obtained by rearing the larvae collected on *M.fruticulosa* and *A.verrucosa*, respectively.

**Figure 7.**
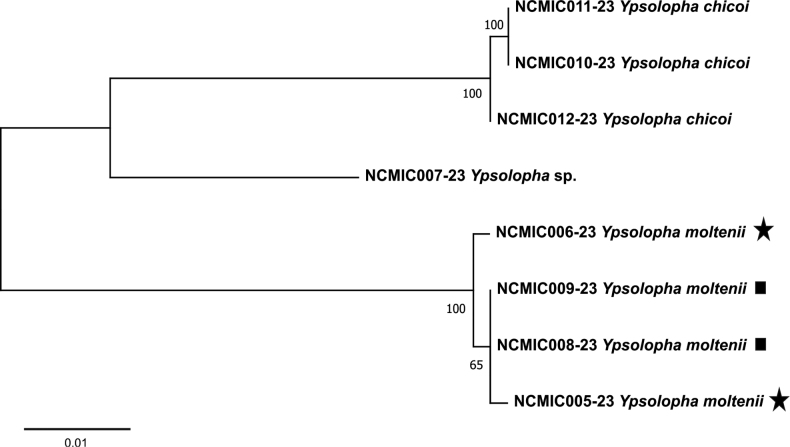
Neighbor-joining tree of DNA barcodes of the genus *Ypsolopha* Latreille, 1796 (Lepidoptera, Ypsolophidae) from the Andes of northern Chile. Stars and squares indicate specimens of *Ypsolophamoltenii* Vargas, 2018 collected on *Adesmiaatacamensis* Meyen and *Adesmiaverrucosa* Meyen (Fabaceae), respectively. Numbers near branches indicate bootstrap support (1,000 replicates).

DNA barcodes were obtained from two females (BOLD Process IDs NCMIC005-23, NCMIC006-23) and two larvae (NCMIC008-23, NCMIC009-23) of *Y.moltenii* from *A.atacamensis* and *A.verrucosa*, respectively; one male (NCMIC007-23) of *Ypsolopha* sp.; and three larvae (NCMIC010-23, NCMIC011-23, NCMIC012-23) of *Y.chicoi*. Genetic divergence (K2P) between individuals of *Y.moltenii* collected on different plants was 0–0.3%. In contrast, genetic divergence of *Y.moltenii* was 7.9–8.1% from *Ypsolopha* sp. and 9.2–9.5% from *Y.chicoi*, while *Ypsolopha* sp. and *Y.chicoi* diverged by 5.9–6.1% (Fig. 7).

### ﻿Material examined

#### ﻿*Ypsolophachicoi* Vargas, 2021

Chile • 1 ♂; Parinacota, Socoroma; [adult emerged] May 2022; H.A. Vargas leg.; ex-larva; *Muehlenbeckiafruticulosa*; [larva collected] April 2022; [genitalia slide] HAV1687 • 2 ♀♀; same data as for preceding; [genitalia slides] HAV1685, HAV1686; all in IDEA.

#### ﻿*Ypsolophamoltenii* Vargas, 2018

Chile • 1 ♂; Parinacota, Putre; [adult emerged] May 2022; H.A. Vargas leg.; ex-larva; *Adesmiaverrucosa*; [larva collected] April 2022; [genitalia slide] HAV1668 • 1♀; same data as for preceding; [genitalia slide] HAV1669 • 2 ♀♀; Zapahuira; [adult emerged] October 2021; H.A. Vargas leg.; ex-larva; *Adesmiaatacamensis*; [larva collected] September 2021; [genitalia slides] HAV1670, HAV1684; [BOLD Process IDs] NCMIC005-23, NCMIC006-23 • 2 ♂♂; same locality as for preceding; [adult emerged] May 2018; ex-larva *Adesmiaatacamensis*; [larva collected] April 2018; [genitalia slides] HAV1179, HAV1671; all in IDEA.

#### ﻿*Ypsolopha* sp.

Chile • 2 ♂♂; Parinacota, Murmuntani; [adult emerged] May 2021; H.A. Vargas leg.; ex-larva; *Adesmiaspinosissima*; [larva collected] April 2021; [genitalia slides] HAV1469, HAV1672; [BOLD Process ID] NCMIC007-23; all in IDEA.

## ﻿Discussion

Detailed knowledge of host ranges is essential to understand abundance and distribution patterns and to plan the conservation of phytophagous lepidopterans (Kozlov 2002; Bassett et al. 2022; Clarke 2022; Vargas 2023). Although the micromoth fauna of the arid, high-elevation area of the western slope of the Andes of northern Chile remains little known, recent surveys have revealed that plants native to this mountain region harbor previously overlooked species whose taxonomic descriptions have become available (e.g. Vargas 2018, 2021). However, some important aspects of the natural history of many recently discovered micromoths, such as their host ranges, remain only partially documented. Thus, surveys for larvae on native plants help to explore the host ranges of the little-known species and to detect unknown ones.

The low genetic divergence between *Y.moltenii* individuals collected on *A.atacamensis* or *A.verrucosa* is similar to conspecific distances previously recorded for *Ypsolopha* micromoths (Ponomarenko 2020), providing further support for the morphological identification. The only host previously recorded for *Y.moltenii* was *A.verrucosa* (Vargas 2018). However, the surveys revealed that this micromoth also uses *A.atacamensis* as a host, while its larvae were not found on other Fabaceae native to the study area, including the congeneric *A.spinosissima*. Since *A.atacamensis* and *A.verrucosa* belong to the same series (*Bracteatae*) of the subgenus Adesmia (Burkart 1967), these data suggest the host range of *Y.moltenii* is restricted to closely related plant species. These results rule out monophagy, suggesting instead oligophagy for *Y.moltenii*.

*Ypsolopha* sp. differs from *Y.moltenii* in morphology and host plant. The genetic divergence between *Ypsolopha* sp. and *Y.moltenii* is also greater than those reported between morphologically similar congeneric species (Corley et al. 2019; Ponomarenko 2020; Corley and Ferreira 2021). Thus, morphology, host plant use, and DNA barcodes confirm that *Ypsolopha* sp. represents a second species of this micromoth genus associated with a member of *Adesmia*. However, as only two males were reared from the larvae collected on *A.spinosissima* in this study, further surveys for larvae on this shrub are needed to obtain females before the formal taxonomic treatment of this micromoth. Although the results suggest that this species is not associated with members the subgenus Adesmia, its larvae should be searched for on additional species of the subgenus Acanthadesmia to assess its host range.

The results of the present study suggest narrow host ranges for *Adesmia*-feeding *Ypsolopha* and highlight the need to explore further the taxonomic diversity of these micromoths in other areas inhabited by these plants. Burkart (1967) recognized two main areas of high species richness for *Adesmia*, the Argentine–Chilean part of the Andes Range and the semiarid area of Argentina east of the Andes. Surveys for larvae in these areas could reveal additional *Ypsolopha* species associated with this highly diverse South American plant genus.

## ﻿Additional information

### Author ORCIDs

Héctor A. Vargas https://orcid.org/0000-0002-5355-3157

### Data availability

All of the data that support the findings of this study are available in the main text.
